# A case report of pneumonia due to non-tuberculous mycobacteria in an immunocompetent patient

**DOI:** 10.11604/pamj.2021.38.412.21501

**Published:** 2021-04-30

**Authors:** Olukemi Adekanmbi, Sulaiman Lakoh

**Affiliations:** 1 College of Medicine, University of Ibadan, Ibadan, Nigeria,; 2 University College Hospital, Ibadan, Nigeria,; 3 College of Medicine and Allied Health Sciences, University of Sierra Leone, Freetown, Sierra Leone,; 4 University of Sierra Leone Teaching Hospitals Complex, Freetown, Sierra Leone

**Keywords:** Non-tuberculous mycobacteria, pneumonia, tuberculosis, immunocompetent, case report

## Abstract

Non-tuberculous mycobacteria are uncommon pathogens in immunocompetent individuals. We report an unusual case of pneumonia with pleural effusion caused by co-infection with two species of non-tuberculous mycobacteria in an immunocompetent man in Nigeria. The case highlights the possibility of the occurrence of a disease caused by these pathogens in an unusual host in a setting where they are rarely isolated as well as the challenges faced in diagnosis.

## Introduction

Nontuberculous mycobacteria (NTM) are ubiquitous in the environment [1,2]. They are known to cause infection most commonly of the lungs but also of skin, soft tissue, and bone [3]. NTM causes pulmonary disease in immunocompromised hosts and those with underlying lung disease [4]. They are a rarely diagnosed cause of pneumonia and pleural effusion in resource-limited settings because of the difficulty in isolating these organisms and also in determining whether or not they are truly pathogenic when identified. We report the case of an immunocompetent 40-year-old man with pulmonary disease caused by 2 species of NTM.

## Patient and observation

A 40-year-old man presented with sudden-onset left-sided chest pain of two days duration. The pain was severe, pleuritic in nature and relieved by non-steroidal anti-inflammatory drugs (NSAIDS). There was a history of self-limited intermittent dry cough over the preceding 3 months and occasional exertional dyspnea. The patient gave a history of recurrent upper respiratory tract infections (URTIs) and a single episode of pneumonia in childhood. There was also a history of URTIs and bronchitis in adulthood for which he was prescribed brief courses of various antibiotics once or twice a year in recent years. Chest radiographs done during these more recent episodes were clear. There was no prior history of previous diagnosis of pulmonary tuberculosis (TB). He had a 10-year history of smoking which stopped 10 years prior to presentation. Physical examination revealed a respiratory rate of 20 cycles per minute, oxygen saturation of 98% on room air and vesicular breath sounds with reduced air entry at the bases. The remainder of the vital signs and physical examination were normal.

**Figure 1:**
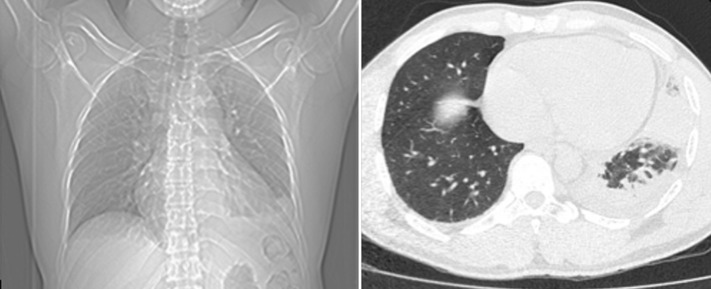
chest radiograph shows a left basal consolidation and small bilateral pleural effusions worse on the left; CT scan of the chest shows consolidation at the left base with a central area of necrosis and bilateral pleural effusions worse on the left

Full blood count showed a total white blood cell count of 4.4x10^9^/L (ref 4.0-11.0 x10^9^ /L) with relative neutropenia and eosinophilia. Other parameters of the full blood count were within normal limits. Erythrocyte sedimentation rate (ESR) was 13 mm in the first hour and the HIV I and II screen was non-reactive. Chest radiograph and computed tomography (CT) scan are shown in Figure 1. A 12-lead electrocardiogram and cardiac troponins showed no evidence of myocardial ischemia or infarction. A 2D echocardiogram showed no regional wall motion abnormalities. D-dimer was within normal limits and angiography of pulmonary vessels showed no filling defect. Flexible bronchoscopy with bronchoalveolar lavage (BAL) showed a hyperemic and inflamed trachea down to the left main bronchus, lingula and left lower lobe with some patchy hyperemic spots. The right bronchopulmonary tree was unremarkable. Routine bacterial culture of BAL fluid revealed no growth and cytology showed features suggestive of chronic inflammation. He was placed on oral moxifloxacin 400mg daily and azithromycin 500mg daily was added to patient´s regimen a few days later. The chest pain improved with antibiotic therapy and the patient was discharged home to complete a 10-day course of oral antibiotics.

**Figure 2:**
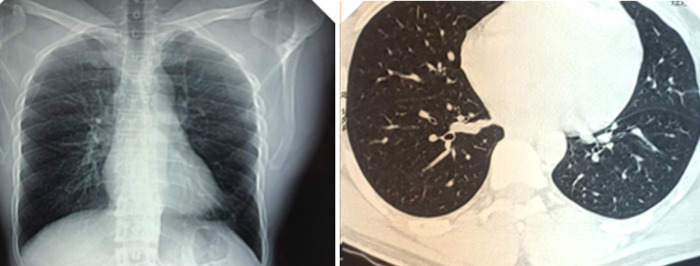
chest radiograph and CT scan at the end of therapy shows no abnormality

Acid-fast bacilli (AFB) smear performed on bronchoalveolar fluid became available after discharge and was positive. The patient was placed empirically on rifampicin, isoniazid, pyrazinamide, ethambutol (RHZE) and pyridoxine for presumed pulmonary TB while awaiting drug susceptibility testing. A few weeks after the commencement of RHZE, the report of the culture of the BAL fluid indicated the isolation of Mycobacterium chelonae subspecie abscessus and Mycobacterium immunogenum. No susceptibility data were made available. Anti-TB therapy was stopped and the patient was placed on Azithromycin and Rifampicin, which he took for about 10 months. He had a repeat bronchoscopy with BAL about 2 months after his initial presentation; all bacterial and mycobacterial cultures and cytology were negative and there were no abnormal findings on bronchoscopy. A chest X-Ray and CT scan at the end of therapy, almost a year after initial presentation showed no abnormality (Figure 2).

## Discussion

The worldwide incidence of NTM is increasing but largely in areas of low prevalence of TB [2,5]. An unusual feature of our patient was co-infection with two species of NTM: Mycobacterium chelonae subspecie abcessus and Mycobacterium immunogenum. NTM co-infection associated with pneumonia and pleural effusion has very rarely been reported in the literature [3,6]. In addition, M. chelonae is a rapidly growing mycobacterium that is rarely isolated in sub-Saharan Africa and like other rapid growers is seen more commonly in East Africa than West Africa. It made up just 1.2% of NTM isolated from pulmonary specimens in a meta-analysis from sub-Saharan Africa [7].

Our patient had true NTM pulmonary disease as he met American Thoracic Society/Infectious Disease Society of America criteria for differentiating colonization from true infection. The criteria are pulmonary symptoms with appropriate radiologic findings AND isolation of NTM from one BAL sample as well as appropriate exclusion of other causes for the clinical and radiologic findings [8]. He presented with pleuritic chest pain; chest pain was reported in 27.3% (the second most common presenting symptom) of patients in a large meta-analysis of NTM isolates in pulmonary samples in sub-Saharan Africa [7]. However, pleural effusion is a rare feature of NTM pulmonary disease [6,8].

In settings such as ours, it is likely that cases of NTM often go undiagnosed as they are usually taken as Mycobacterium tuberculosis (MTB) when associated with sputum AFB smear positivity and because they are also not detectable by the XpertMTB Rif® test. These are the two investigations widely available for diagnosis of Mycobacterial infection in resource limited settings as mycobacterial cultures and molecular studies (other than the XpertMTB Rif) are not routinely available. Of note, our patient did not have the XpertMTB Rif® test because it was not available at our facility at the time he presented. NTM disease is more likely to be associated with sputum AFB negative specimens; however, sputum AFB negativity with a suggestive clinical syndrome does not typically prompt investigating for NTM rather, it is often treated as smear negative TB with anti-TB medications [9].

There is limited capacity for diagnosis of NTM in RLS with the focus on mycobacterial disease diagnostics being placed on TB. Our patient was treated initially with moxifloxacin, azithromycin and anti-tuberculous drugs all of which have some activity against many strains of NTM. Most patients with a similar presentation may not have access to extensive investigations such as this patient had and would likely receive some or all of the above antibiotics empirically with some clinical improvement. Our patient did have some risk factors for NTM disease. He is a former smoker, had a history of recurrent respiratory tract infections in childhood and as an adult. He, however, did not have some of the more classic risk factors associated with the disease in Europe and North America such as advanced age, chronic and/or structural lung disease and current smoking [5]. In sub-Saharan Africa, risk factors for NTM pulmonary disease include HIV positive status and prior treatment for pulmonary TB and a younger age distribution than is seen in Europe and North America [7].

## Conclusion

It is reasonable to consider NTM as causative organisms in pneumonia that do not present typically in immunocompetent hosts and evaluate them accordingly. Patients presumptively diagnosed with pulmonary TB or diagnosed by AFB smear alone should also be considered for evaluation for NTM disease as should those with smear negative disease. Epidemiology and risk factors for NTM in various geographical regions, particularly in sub-Saharan Africa should be further studied and clearly understood to provide data to support the screening for NTM disease. Capacity to identify NTM down to the species level and perform drug susceptibility testing on the isolates in resource limited settings need to be strengthened.
